# Task-dependent cross-frequency neural coupling during postural perturbation: insights from EEG-based assessment in elite freestyle aerial skiers

**DOI:** 10.3389/fphys.2025.1700523

**Published:** 2025-12-18

**Authors:** Yuqi Cheng, Ao Fu, Youcai Guo, Jie Gao, Yongxia Chen, Qianrong Qi, Feng Guo, Xin Wang

**Affiliations:** 1 School of Exercise and Health, Shenyang Sport University, Shenyang, China; 2 Department of General Education, Guangdong ATV College of Performing Arts, Guangdong, China; 3 Winter Olympic Academy, Harbin Sport University, Harbin, China

**Keywords:** cross-frequency coupling (CFC), EEG, phase-amplitude coupling (PAC, ), balance tasks, freestyle skiers

## Abstract

**Background:**

Cross-frequency coupling (CFC), particularly phase–amplitude coupling (PAC), reflects hierarchical interactions between neural oscillations and plays a critical role in sensorimotor integration. However, its functional relevance during balance control under sensory perturbations remains insufficiently understood.

**Objective:**

This study aimed to investigate PAC characteristics during postural control tasks of varying difficulty in elite freestyle aerial skiers versus non-athlete controls.

**Methods:**

EEG signals and center of pressure (COP) data were recorded from participants performing six standing balance tasks on stable and unstable surfaces. Postural control was assessed using center of pressure data, which represent the point location of the body’s vertical ground reaction force vector and are commonly used to quantify sway and balance performance during stance. Mean Vector Length Modulation Index (MVLmi) and PAC analyses were applied to assess oscillatory interactions.

**Results:**

Surface instability significantly modulated PAC strength across frequency bands (*P* < 0.05). Athletes exhibited task-specific enhancements in alpha-gamma and delta-gamma coupling during single-leg and double-leg stance. These coupling patterns were more spatially localized and showed trends consistent with superior postural control. In contrast, non-athletes showed widespread PAC increases under perturbation, but with less effective balance performance. Hemispheric asymmetries were observed during single-leg stance: athletes demonstrated contralateral dominance during right-leg tasks and ipsilateral coupling shifts during unstable left-leg stance, indicating dynamic lateralized control shaped by training. Across conditions, athletes showed higher PAC strength and lower sample entropy, reflecting more efficient and adaptable cortical strategies for postural regulation.

**Conclusion:**

PAC strength is closely linked to postural performance and varies with task complexity and surface condition. These findings highlight the role of training-induced neuroplasticity in modulating cortical dynamics for balance control, offering new insights for targeted neuromodulatory interventions and neurofeedback-based training strategies.

## 1 Introduction

Human balance is a complex neurophysiological function that depends on the continuous integration of multisensory input and the precise coordination of motor output. The central nervous system (CNS) maintains postural stability by processing information from the visual, vestibular, and proprioceptive systems. Disruption in any of these modalities can impair balance and increase postural sway (Henry and Baudry, 2019; Horak and Hlavacka, 2001). Although isolating vestibular contributions in experiments remains challenging, they act in concert with visual and proprioceptive signals to sustain upright posture (Keyvanara et al., 2021).

Recent electrophysiological studies have shown that cortical activity is highly responsive to postural challenges. Oscillations in specific frequency bands—particularly increased theta power in frontal and central regions—are associated with elevated cognitive demand and the need to resolve sensorimotor conflicts during balance perturbations (Chen X. P. et al., 2021; Tsai et al., 2022). For instance, theta activity in the precentral cortex rises during complex postural tasks, reflecting the additional processing required to coordinate movement and maintain stability (Peterson and Ferris, 2018; Stokkermans et al., 2023; Mierau et al., 2015). These findings emphasize the dynamic nature of neural adaptation in postural control, highlighting the brain’s role in adjusting to varying stability demands.

Historically, studies of brain oscillations focused on the roles of individual frequency bands. Since Berger’s discovery of the alpha rhythm (8–12 Hz) in 1929 (Berger, 1929), low-frequency oscillations have been linked to motor control and sensory input, whereas high-frequency activity has been associated with localized neural processing. More recently, research has shifted toward understanding how distinct frequencies interact. Low-frequency rhythms, often involved in both external sensory-motor functions and internal cognitive processes such as decision-making and memory (Schroeder and Lakatos, 2009), are now known to interact with high-frequency signals that are topographically localized. Cross-frequency coupling (CFC)—especially phase–amplitude coupling (PAC)—has emerged as a mechanism for integrating information across spatial and temporal scales. Although CFC is considered important for coordinating sensory, motor, and cognitive functions, its specific contribution to balance control remains underexplored (Buzsáki and Draguhn, 2004). In parallel, EEG power spectral density (PSD) analysis has become a standard tool for assessing frequency-specific brain responses to postural demands (Peterson and Ferris, 2018). For example, Hülsdünker et al. (2015) found that increased task difficulty was accompanied by greater frontal theta power, suggesting enhanced sensorimotor integration during balance regulation. Likewise, Slobounov et al. (2006) reported elevated theta and reduced alpha power during postural instability induced by surface perturbation. These results support the use of PSD as a sensitive indicator of cortical adaptation in response to balance challenges.

CFC describes the interactions between neural oscillations of different frequencies and is widely recognized as playing a crucial role in brain functions such as computation, information transmission, and learning (Salimpour and Anderson, 2019). Early studies identified that distinct frequency bands predominantly underlie specific types of brain activity and respond selectively to sensory, motor, and cognitive stimuli (Engel et al., 2001). Within this theoretical framework, CFC has been categorized into several forms, including amplitude–amplitude coupling (AAC), also known as comodulation (Yeh et al., 2016); phase–phase coupling (PPC), which involves harmonic synchronization (Scheffer-Teixeira and Tort, 2016); and the most extensively studied form, PAC. Among these, PAC has drawn increasing attention for its critical role in regulating neural dynamics, particularly in pathological conditions such as epilepsy (Hyafil et al., 2015; Malladi et al., 2018).

PAC occurs when the amplitude of high-frequency neural activity in one brain region is modulated by the phase of low-frequency oscillations, either within the same area or across regions. Among the most commonly studied forms are theta–gamma and alpha–gamma coupling, which have been used to examine interactions between distinct cognitive systems (Canolty and Knight, 2010). PAC modulation is often driven by dominant low-frequency rhythms that are characteristic of specific brain areas and behavioral contexts. For example, Osipova et al. (2008) showed that gamma-band power was synchronized with the alpha, rather than the theta, phase. Similarly, Cohen et al. (2009) identified alpha–gamma coupling in the human ventral thalamus. As a mechanism for linking neural activity across spatial and temporal scales, PAC facilitates inter-regional communication and coordination. Robust CFC patterns have been reported in several brain structures—including the neocortex, hippocampus, and basal ganglia—during task execution, with coupling frequencies varying by region and task. Moreover, multiple coupling modes may coexist depending on functional demands.

Although CFC has been increasingly studied as a mechanism of cognitive integration, its role in postural control-especially under conditions of sensory perturbation-remains poorly understood. Among the various forms of CFC, PAC is of particular interest because it captures hierarchical interactions between the phase of low-frequency and the amplitude of high-frequency brain rhythms. Yet, it is not fully known how PAC supports balance regulation, or how this coupling adapts to task complexity and sensory challenges. Studying brain activity during challenging standing tasks in elite athletes is especially valuable, as this population operates near the upper limits of human sensorimotor performance, where subtle neural adaptations become most apparent (Nakata et al., 2010). Previous research has shown that elite athletes exhibit distinctive cortical activation patterns during balance regulation (Del Percio et al., 2009). Examining PAC in this cohort during progressively challenging standing tasks may therefore elucidate how hierarchical neural coordination supports elite-level balance regulation and rapid compensatory adjustments. Based on this rationale, the present study aimed to quantify PAC in elite athletes during balance tasks of increasing difficulty and sensory perturbation, thereby characterizing the contribution of cross-frequency neural interactions to high-level postural control.

Building upon this framework, we hypothesized that (1) neural coupling strength would differ across brain regions, reflecting region-specific adaptations for postural control, and (2) external perturbations such as unstable support surfaces would modulate PAC strength, revealing the context-dependent nature of neural coordination during balance tasks.

## 2 Methods

### 2.1 Participants

This study recruited 14 elite freestyle aerial skiing athletes, each with more than 5 years of professional training experience, as the experimental group. All athletes were ranked within the top eight in either the Winter Olympics or the World Cup. The participants had a mean height of 167.95 ± 8.70 cm, body weight of 63.11 ± 10.28 kg, and mean age of 24.63 ± 5.01 years. A control group of ten healthy adults who did not participate in regular physical training (less than three sessions per week) was also recruited. The average height, weight, and age of this group were 172.86 ± 5.5 cm, 72 ± 9.65 kg, and 25.35 ± 2.4 years. All participants were confirmed to be right-leg dominant using a preferred leg test involving a ball-kick task, as required by the single-leg balance protocol.

Prior to participation, each subject completed a detailed medical history questionnaire to screen for musculoskeletal, neurological, or systemic conditions that could interfere with single-leg balance. None reported lower limb injuries within the past 6 months. The research protocol received ethical approval from the Ethics Committee of Shenyang Sport University under approval number 2018 (09). All methods described in this study were conducted in accordance with the relevant guidelines and regulations as outlined in the Declaration of Helsinki.

### 2.2 Material and procedure

#### 2.2.1 Equipment

Postural data were collected using a portable balance platform (Humac Balance, United States; 65 × 40 cm, 100 Hz sampling rate), which provided a stable surface condition. To induce proprioceptive instability, a foam pad of the same dimensions (65 × 40 cm, 50 mm thick) was placed on the platform. The center of pressure (COP) was recorded under both stable and unstable conditions.

Cortical activity was simultaneously recorded using a 64-channel EEG system (eego™ mylab, ANT Neuro, Netherlands). Participants wore a standard EEG cap with conductive gel applied to all electrodes. Electrode impedance was maintained below 5 kΩ throughout data acquisition. EEG signals were sampled at 2000 Hz and filtered online with a 0.1–100 Hz bandpass. The ground electrode was positioned between FPz and Fz, and CPz served as the reference. This midline location was selected to reduce hemispheric bias and is frequently used in postural control and motor-related EEG studies (Castillo-Barnes et al., 2024), in accordance with the international 10–20 system.

#### 2.2.2 Testing procedure

Participants were asked to remove their shoes and socks and stand barefoot on the platform, maintaining an upright trunk posture with minimal body sway. All tests were conducted in a quiet, temperature-controlled laboratory to minimize external distractions. Participants placed their hands on their hips and positioned both feet symmetrically, with the forefoot edges aligned to the marked reference line on the platform. The reference line defined a standardized stance angle of approximately 45°between the feet, a configuration commonly adopted in postural control research to provide a natural standing posture and ensure consistency across participants.

Visual fixation was maintained on a 5 cm white circle positioned at eye level on a wall 3 m ahead. Participants were instructed to avoid unnecessary movements and to keep their hips and knees slightly flexed during all trials.

Each participant completed a proprioceptive interference and single-leg stance test consisting of six static postural conditions. These conditions have shown high test–retest and inter-rater reliability in healthy young adults (Hof, 2007). The specific conditions were as follows:

T1 (EOF): Bipedal stance on an unstable surface.

T2 (EO): Bipedal stance on a stable surface.

T3 (LOF): Left-leg stance on an unstable surface.

T4 (LO): Left-leg stance on a stable surface.

T5 (ROF): Right-leg stance on an unstable surface.

T6 (RO): Right-leg stance on a stable surface.

Each condition lasted 30 s, with a 30-s seated rest between trials. EEG signals and plantar pressure data were recorded simultaneously to assess neural activity and balance control.

Several procedures were followed to minimize potential confounds. All testing took place during the athletes’ off-season, and participants were asked to avoid strenuous physical activity for 24 h prior to testing. They were also instructed to maintain their usual daily routines while avoiding behaviors that could influence mood or physiological state. All assessments were conducted in a temperature-controlled laboratory under standardized conditions to ensure consistency and data quality.

### 2.3 Data processing

#### 2.3.1 EEG data preprocessing

EEG preprocessing was performed using the EEGLAB toolbox in MATLAB, following a standardized and widely accepted pipeline. Raw signals were filtered with a fourth-order Butterworth bandpass (1–40 Hz) to remove slow drifts and high-frequency noise. A notch filter at 50 Hz (48–52 Hz) was applied to eliminate line noise. All signals were re-referenced to the common average to enhance baseline consistency.

Artifact correction involved multiple steps to address ocular, muscular, and movement-related noise. Independent Component Analysis (ICA) was applied to isolate and remove artifactual components. These were identified based on their temporal signatures (transient bursts, stereotyped rhythmic activity), spatial topographies (fronto-orbital or temporalis localization), and spectral features showing non-physiological power concentrations (>50 Hz or <1 Hz).

Following ICA, amplitude-based thresholding was applied to identify and remove residual high-amplitude artifacts. EEG data were segmented into 2-s epochs, and any segment containing voltage fluctuations exceeding ±80 μV was automatically rejected. To ensure data integrity, automatic classification was supplemented with visual inspection. On average, 2.67 ± 0.45 components were removed per dataset. Residual segments contaminated by noise or with incomplete recordings were excluded. Only artifact-free, high-quality EEG epochs were retained for subsequent PAC analysis.

#### 2.3.2 Mean vector length modulation index, MVLmi

Given the variability of cross-frequency interactions during postural control, this study specifically focused on PAC-a subtype of CFC-to assess the coordination between neural oscillations across different frequency bands. The EEG frequency bands were defined as Delta (1–3 Hz), theta (4–7 Hz), alpha (8–12 Hz), beta (13–30 Hz), and gamma (30–40 Hz). During processing, the low-frequency bandwidth (phase) was set to 1–29 Hz, and the high-frequency bandwidth (amplitude) was set to 7–40 Hz. After preprocessing, the Hilbert transform was applied to calculate the instantaneous phase information for the low-frequency range and the instantaneous amplitude information for the high-frequency range. These results were subsequently used for CFC analysis.

Subsequently, this study employed the method proposed by Canolty and Knight (2010) to calculate CFC. The MVLmi depends on generating a composite signal $Z_{t}$ in the complex plane, as shown in the formula.

$$Z_{t}=A_{env}\cdot e^{i\cdot \Phi_{phase,t}}$$

$Z_{t}$ :The value of the composite signal at time t, $A_{env}$ : instantaneous amplitude of a high-frequency signal at time t, i imaginary unit, $\Phi_{phase,t}$ instantaneous phase of a low-frequency signal at time t.

The value of the MVLmi is computed by taking the average length of $Z_{t}$ across all time points.

$$MI=\left|mean\left(z\right)\right|$$

$mean\left(z\right)$ , average of the composite signal $Z_{t}$ across all time points is computed, and this value is utilized to analyze phase-amplitude coupling.

The formula for calculating the average magnitude of the composite signal is as follows:

$$MVLmi_{abs}=abs\left(z_{mean}\right)z_{mean}$$

$$MI_{p}=\frac{\sum_{i=1}^{n_{surr}}MI_{surr_{i}}>MI+1}{n_{surr}+1}$$

${MI}_{{surr}_{i}}$ represents the MVL-MI value obtained from the i simulation. The calculation of the p-value involves determining the proportion of simulated This ratio provides the MVLmi p-value. If the calculated p-value for the observed MVLmi is small (*P* < 0.05), then the observed MVLmi is considered statistically significant.

In this study, the randomization process involves randomly dividing the signal into two segments. This randomization method simulates the situation where signal segmentation occurs under random conditions, allowing for an exploration of the sensitivity of MVLmi to signal segmentation.

$$normalized_{MI}=\frac{MI-\mu }{\sigma }$$

μ represents the mean of the MVLmi obtained from 100 randomizations, and σ is the standard deviation.

#### 2.3.3 Sample entropy (SE)

Early studies have shown that Sample Entropy (SE) can quantify the regularity or predictability of COP time series data collected under different test conditions or across different experimental groups (Kirchner et al., 2012). By expanding the dimensionality range of all one-dimensional COP data from the measurement results, calculating the absolute value of the maximum difference between two vectors, and comparing it with a threshold, more regular features can be discovered. In this study, we processed the measured COP data, where the data length N was 3,000 points (sampling frequency of 100 Hz, sampling time of 30 s). Through calculation, we expanded the complex and variable original one-dimensional COP data within the time series into more regular two-dimensional and three-dimensional data, thereby obtaining a more regular SEn value (Ahmadi et al., 2018).

SE (m, r, N) refers to the negative natural logarithm of the conditional probability that two sequences similar for m points remain similar at the next point, m+1 , where self-matches are not included in the probability calculation. Apart from removing self-matches, SE has been proven to be independent of data length and capable of providing more meaningful results (Richman and Moorman, 2000).

The formula is as follows:

For a time series $\left(x_{i}\right)=\left(x_{1,}x_{2,}x_{3,}\cdots x_{n}\right)$ , composed of *N* data points, we form a sequence of m-dimensional vectors $y_{i\left(m\right)}$ :

$$y_{i\left(m\right)}=\left(x_{i},x_{i+1},\ldots ,x_{i+m-1}\right),i=1,2,\ldots ,N-m+1$$

Next, without considering self-comparisons, we find matching templates by comparing the polynomial distance to $B_{i}$ a predetermined threshold r . Then, we construct a variable which is the number of pairs that satisfy the aforementioned condition:

$$B_{i}^{m}\left(r\right)=\frac{1}{N-m-1}\sum_{j=1,j\neq 1}^{N-m}\Theta \left(r-\|\left\|y_{j}\left(m\right)-\left.y_{i}\left(m\right)\right\|\|\infty \right.\right)$$

In the above equation $\|\left\|y_{j}\left(m\right)-\left.y_{i}\left(m\right)\right\|\|\infty =\max0\leq \right.k\leq m-1\left|x_{j+k}-\left.x_{i+k}\right|\right.$ representing the maximum distance between two vectors, which is the absolute value of the maximum difference between the two vectors; Θ is the unit step function used to determine whether the maximum distance between two vectors is less than or equal to the threshold r ;

We calculate two quantities, $B\left(m\right)$ and $A\left(m\right)$ :

$$B^{m}\left(r\right)=\frac{1}{N-m}\sum_{i=1}^{N-m}B_{i}^{m}\left(r\right)$$

Increase the dimension to m+1 , constructing a set of vectors with dimension m+1 . Repeat the above steps to calculate $A_{i}^{m}\left(r\right)$ and $A^{m}\left(r\right)$ :

$$A_{i}^{m}\left(r\right)=\frac{1}{N-m-1}\sum_{j=1,j\neq 1}^{N-m}\Theta \left(r-\|\left\|y_{i}\left(m+1\right)-y_{i}\left.\left(m+1\right)\right\|\right.\|\infty \right)$$

$$A^{m}\left(r\right)=\frac{1}{N-m}\sum_{i=1}^{N-m}A_{i}^{m}\left(r\right)$$

Finally, according to SE (Montesinos et al., 2018), with m = 2 and r = 0.1SD, the following formula is obtained for the negative logarithm of the conditional probability of the sequence of data dimensions:

$$SampleEn\left(m,r,N\right)=-\log\left(\frac{A^{m}\left(r\right)}{B^{m}\left(r\right)}\right)$$

Calculate the Euclidean norm of the entropy values obtained for the anterior-posterior (AP) direction and medial-lateral (ML) direction samples, evaluating a comprehensive indicator of athletes’ balance abilities.

$$Euclidean_{SE}=\sqrt{ML_{SE}^{2}+AP_{SE}^{2}}$$

#### 2.3.4 Sway velocity

Sway velocity was included as an additional behavioral measure of postural stability to complement SE. Whereas SE reflects the temporal irregularity of COP fluctuations, sway velocity captures the overall rate of COP displacement and is widely used as a sensitive indicator of balance performance under both stable and perturbed conditions.

For each trial, sway velocity was calculated from the COP time series recorded by the force platform. Instantaneous COP displacement between successive samples was computed in the anterior-posterior and medial-lateral directions and then divided by the corresponding sampling interval. The mean sway velocity for each task was obtained by averaging the resultant COP velocity across the entire trial duration. Higher sway velocity values indicate greater postural instability and reduced efficiency of balance control.

$$SwayVelocity=\frac{1}{T}\sum_{i=1}^{N-1}\frac{\sqrt{{\left(x_{i+1}-x_{i}\right)}^{2}+{\left(y_{i+1}-y_{i}\right)}^{2}}}{\Delta t}$$

where: $x_{i},y_{i}$ : COP coordinates at the i -th sampling point; Δt :sampling interval (100-Hz device); N : total number of samples; $T=\left(N-1\right)\Delta t$ .

### 2.4 Data analysis

**FIGURE 1:**
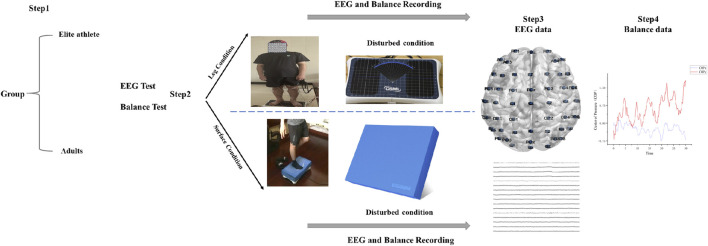
Experimental set-up. In Step 1, We selected 14 elite freestyle skiing aerials athletes and 10 adults. Step 2 involves conducting two experiments on all participants: first, 30-s electroencephalogram (EEG) and balance recording test during various support leg conditions, and second, a 30-s test under varying surface conditions. Throughout this process, detailed EEG and Balance data for all participants are meticulously recorded. In step 3, Electrode positions, providing examples of electrodes on the top of the head for all participants. The coordinates of these electrodes are referenced from the node file of the 10/20 EEG system based on Koessler’s research (Koessler et al., 2009). In step 4, Center of Pressure data (anterior-posterior and mediolateral) were recorded to further analyze balance.

All statistical analyses were performed using MATLAB R2022a, and spatial brain maps were visualized with the BrainNet Viewer toolbox (Figure 1). Group-level comparisons of mean vector length modulation index (MVLmi) values and balance performance across task conditions were conducted using independent-samples *t*-tests, whereas within-group effects of support surface stability (T1 vs. T2, T3 vs. T4, T5 vs. T6) were assessed using paired-samples *t*-tests after standardizing MVLmi values across all electrodes. Bonferroni correction was applied to adjust for multiple comparisons and control the family-wise error rate. Statistical significance was set at *p* < 0.05, and highly significant differences were defined as *p* < 0.01.

To further examine task-dependent variations, pairwise comparisons were performed among all six postural tasks within each group. These analyses evaluated changes in PAC strength and balance performance associated with increasing postural complexity and different stance conditions.

Given the relatively small sample size, a non-parametric permutation test was additionally performed to validate the robustness of group-level comparisons. This method does not assume normality and provides more reliable inference under limited sample conditions. Permutation-derived *p*-values were subjected to the same Bonferroni correction to adjust for multiple comparisons. Results from permutation tests were used to cross-validate the findings from parametric tests.

## 3 Results

### 3.1 EEG signal characteristics in time and frequency domains

**FIGURE 2:**
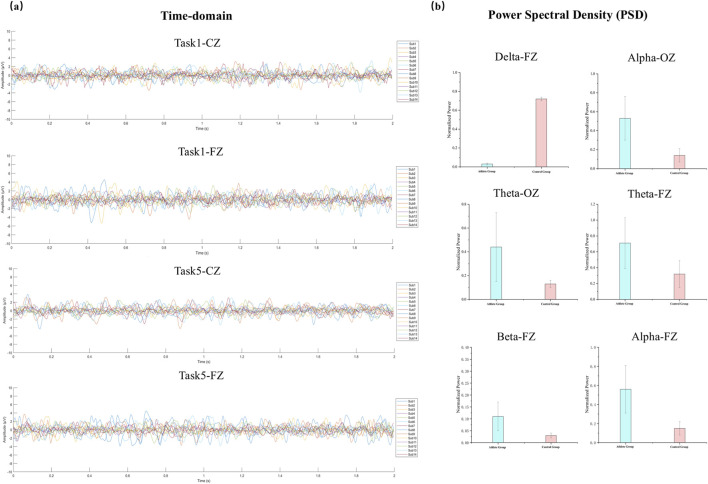
EEG waveforms and PSD comparisons between groups. **(a)** Presents time-domain EEG waveforms from two representative tasks (Task1 and Task5) at the FZ and CZ electrodes. Signals from all participants are overlaid to demonstrate the temporal consistency and variability across subjects. **(b)** Normalized PSDs for elite athletes and non-athlete controls, showing frequency bands with statistically significant differences (*P* < 0.05).

To assess the quality of the EEG recordings and to inform the selection of frequency bands for the subsequent PAC analysis, both time-domain and frequency-domain characteristics of the signals were examined under representative task conditions. Figure 2a illustrates sample EEG waveforms from the Fz and Cz electrodes during Task 1 and Task 5. Each line represents the average amplitude across all 2-s epochs for an individual participant. The waveforms showed consistent temporal structure and oscillatory dynamics across subjects, with no evidence of significant artifacts or channel failure, confirming the overall reliability of the data.

As shown in Figure 2b, normalized PSD values were compared between groups at selected electrodes (Fz and Oz) where significant differences were observed. Relative to the control group, elite athletes displayed significantly greater alpha power at both Fz and Oz, as well as elevated theta and beta power at Fz (*p* < 0.05).

### 3.2 MVLmi of all electrodes

**FIGURE 3:**
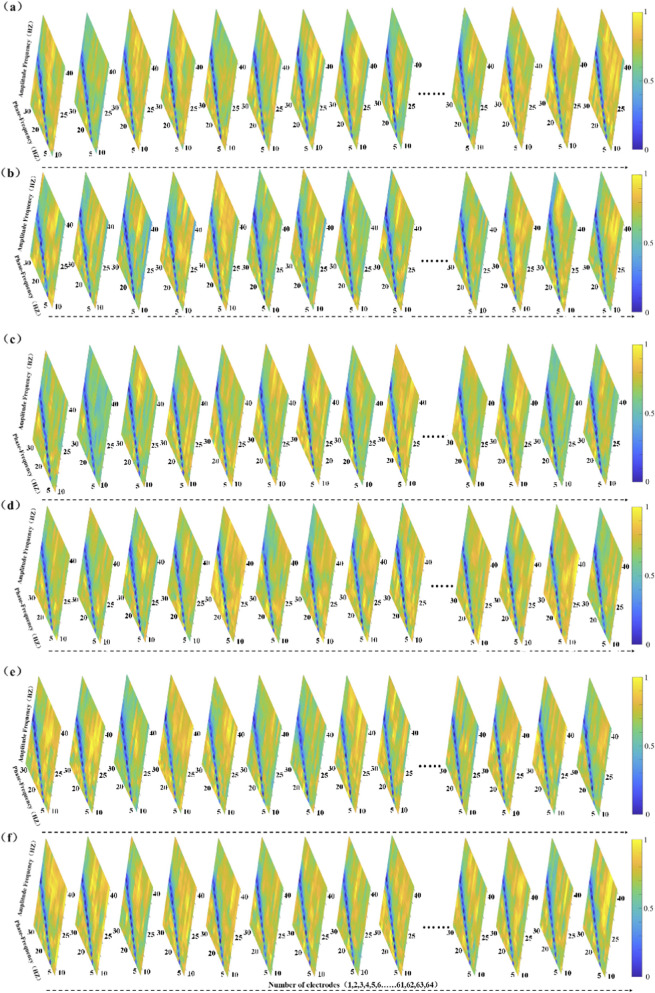
The figure panels **(a–f)** present the Modulation Index-based Cross-Frequency Coupling (MVLmi) results across 13 electrodes for a representative athlete performing six distinct balance tasks (T1-T6). In each panel, the horizontal axis represents the phase-frequency range (capturing low-frequency oscillations such as δ, θ, α, β), while the vertical axis corresponds to the amplitude-frequency range (capturing high-frequency components, primarily in the γ band). This creates a 15 × 22 matrix of phase-amplitude coupling (PAC) data points.

Figure 3 illustrates PAC heatmaps for the elite athlete group across six static balance tasks. Each heatmap depicts a coupling matrix spanning 15 phase-frequency bands and 22 amplitude-frequency bands, reflecting interactions between low- and high-frequency oscillations under varying postural demands.

The results reveal distinct coupling patterns and spatial distributions across different task conditions, indicating that athletes modulate oscillatory activity in task-specific ways. Notably, only data from the athlete group are presented here. Although the control group completed the same tasks, their PAC distributions are not shown. All analyses were performed using identical preprocessing and statistical procedures across both groups.

### 3.3 Cross-frequency coupling differences in support surface interference

#### 3.3.1 Dual-leg standing (T1 vs. T2)

**FIGURE 4:**
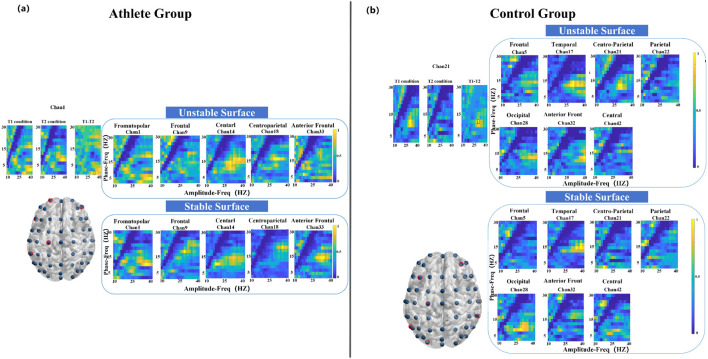
Electrode locations (lower left) and MVLmi computed for T1 and T2 balance tasks at a subset of channels electrodes (*P* < 0.05). **(a)** Represents the results for athletes, and **(b)** represents the results for adults. In **(a)**, the difference in Channel 1 is magnified in the upper left. The MVLmi maximum of T1 and T2 (32-Hz amplitude, 2-Hz phase) is highlighted with a dotted box (upper left). In **(b)**, the difference in Channel 21 is magnified in the upper left. The MVLmi maximum of T1 and T2 (28-Hz amplitude, 12-Hz phase) is highlighted with a dotted box (upper left).

As illustrated in Figure 4a, the athlete group exhibited significant PAC differences (*P* < 0.05) at five electrode sites, primarily located in the frontopolar, frontal, anterior frontal, and central regions. In contrast, the control group showed significant coupling changes at seven sites, encompassing broader cortical areas including the frontal, temporal, parietal, and occipital lobes. Notably, the most prominent modulation in the athlete group occurred at the frontopolar site, with enhanced coupling between 2 Hz (phase) and 32 Hz (amplitude). The control group, on the other hand, demonstrated a peak difference at 12 Hz (phase) and 28 Hz (amplitude), as depicted in Figure 4b.

#### 3.3.2 Left-leg standing (T3 vs. T4)

**FIGURE 5:**
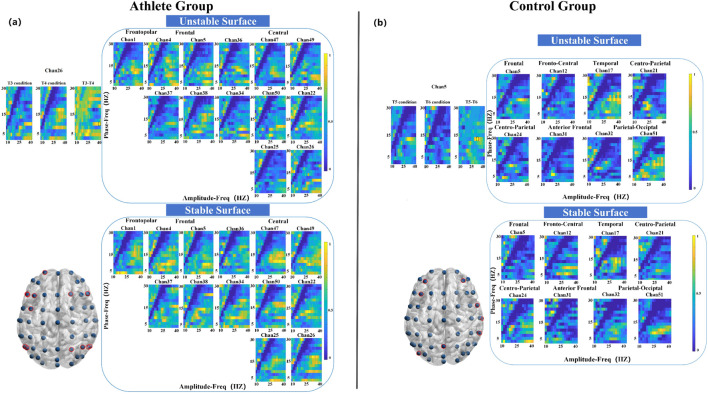
Electrode locations (lower left) and MVLmi computed for T3 and T4 balance tasks at a subset of channels electrodes (*P* < 0.05). **(a)** Represents the results for athletes, and **(b)** represents the results for adults. In **(a)**, the difference in Channel 26 is magnified in the upper left. The MVLmi maximum of T3 and T4 (38-Hz amplitude, 10-Hz phase) is highlighted with a dotted box (upper left). In **(b)**, the difference in Channel 5 is magnified in the upper left. The MVLmi maximum of T3 and T4 (32-Hz amplitude, 14-Hz phase) is highlighted with a dotted box (upper left).

During the left-leg standing task, the athlete group exhibited significant PAC alterations across 13 electrode sites, with pronounced effects in the parietal cortex (Figure 5a). The strongest coupling difference was observed at a phase frequency of 10 Hz and an amplitude frequency of 38 Hz. In comparison, the control group showed significant changes at eight electrode sites distributed across seven cortical regions, with the most pronounced PAC occurring in the frontal cortex at 14 Hz (phase) and 32 Hz (amplitude) (Figure 5b).

#### 3.3.3 Right-leg standing (T5 vs. T6)

**FIGURE 6:**
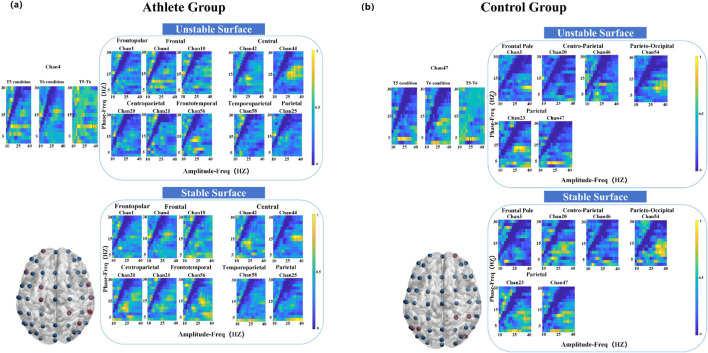
Electrode locations (lower left) and MVLmi computed for T5 and T6 balance tasks at a subset of channels electrodes (*P* < 0.05). **(a)** Represents the results for athletes, and **(b)** represents the results for adults. In **(a)**, the difference in Channel 4 is magnified in the upper left. The MVLmi maximum of T5 and T6 (32-Hz amplitude, 10-Hz phase) is highlighted with a dotted box (upper left). In **(b)**, the difference in Channel 47 is magnified in the upper left. The MVLmi maximum of T5 and T6 (22-Hz amplitude, 4-Hz phase) is highlighted with a dotted box (upper left).

Compared to the left-leg condition, fewer significant differences were observed during right-leg standing. Athletes showed coupling differences in 10 electrodes primarily localized to the frontal and central areas, with peak PAC at 10 Hz phase and 32 Hz amplitude (Figure 6). The control group exhibited coupling differences across six electrodes, spanning the frontal, parietal, and occipital cortices, with key PAC activity at 4 Hz phase and 22 Hz amplitude.

### 3.4 Coupling characteristics between ipsilateral and contralateral hemispheres

**FIGURE 7:**
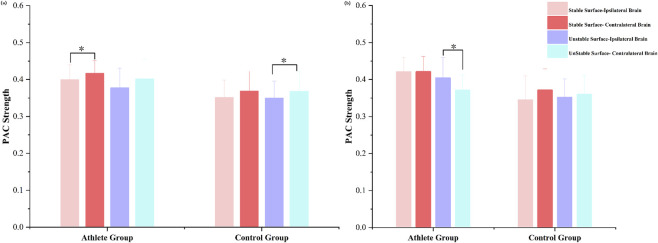
Bilateral hemispheric PAC strength in athletes and adults during **(a)** right-leg stance and **(b)** left-leg stance. Mean PAC values are shown for different stance conditions (T3–T6 tasks). *P* < 0.05 indicates significant differences within each group.

To investigate lateralized neural responses during unilateral stance, we analyzed PAC strength between the ipsilateral and contralateral hemispheres under both left- and right-leg support conditions (Figure 7).

During right-leg stance, all participants demonstrated stronger PAC in the hemisphere contralateral to the supporting limb, regardless of surface stability. However, the magnitude of this hemispheric asymmetry varied between groups and surface conditions. Specifically, elite athletes exhibited significantly greater contralateral-to-ipsilateral PAC differences under stable support conditions (*P* < 0.05), whereas the control group showed more pronounced asymmetry on the unstable surface (*P* < 0.05).

A different pattern emerged during left-leg stance. While the control group maintained stronger contralateral PAC across both surface types, the athlete group exhibited a notable shift in coupling direction under unstable conditions. In this context, PAC strength was significantly greater in the ipsilateral hemisphere compared to the contralateral side (*P* < 0.05).

### 3.5 PAC strength and balance performance

**FIGURE 8:**
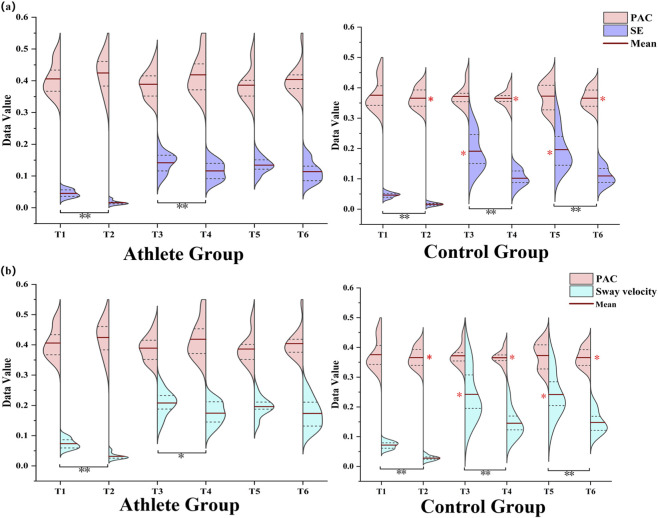
**(a,b)** show the relationship between PAC strength and balance performance, assessed using SE and sway velocity, across all six postural conditions (T1–T6) for both groups. The color-coded plots represent the relative magnitudes of PAC, SE, and sway velocity observed under each task condition.

In the control group, both SE and sway velocity were significantly higher on unstable surfaces than on stable ones across all postural tasks (*P* < 0.05), indicating reduced postural stability in response to proprioceptive disturbance. Among elite athletes, significant increases in SE and sway velocity were observed during bipedal and left-leg stances under unstable conditions (*P* < 0.05), whereas performance during right-leg stance (dominant limb) remained relatively stable (Figure 8).

Further between-group comparisons revealed that athletes exhibited significantly greater PAC strength than controls during the T2 (bipedal-stable), T4 (left-leg-stable), and T6 (right-leg-stable) tasks (*P* < 0.05). In contrast, during the T3 and T5 tasks, athletes achieved lower SE values and sway velocity compared with controls (*P* < 0.05), indicating better postural stability under these conditions.

**FIGURE 9:**
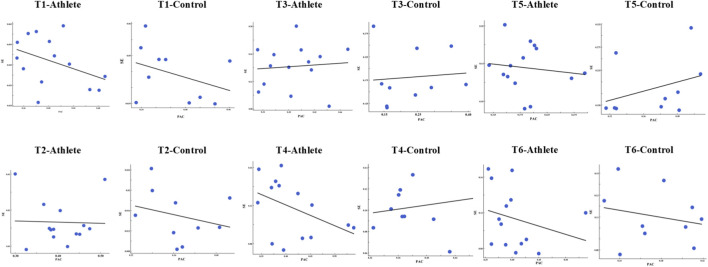
Scatterplots illustrating the relationship between (PAC strength and SE across the six postural tasks (T1–T6) in the athlete and control groups. Each panel displays individual data points (blue dots) and the corresponding linear regression fit (black line).

Across task conditions, the association between PAC strength and postural entropy showed task and group dependent variability rather than a uniform pattern. In several tasks-particularly in the athlete group (T1, T4, and T6)-a clear negative trend was observed, whereby stronger cross-frequency coupling tended to accompany lower SE values. In other tasks, however, the relationship was weaker or showed no consistent direction (Figure 9).

## 4 Discussion

The present study provides new evidence for the neural mechanisms supporting postural control under proprioceptive challenge. In response to unstable surfaces, elite athletes exhibited stronger and more spatially specific PAC, particularly within prefrontal and parietal regions involved in top-down regulation and sensorimotor integration. These task-dependent coupling patterns—most prominently in the alpha–gamma and delta–gamma ranges—suggest that elite performers rely on efficient, context-sensitive neural strategies to maintain balance when sensory reliability decreases.

### 4.1 Effects of proprioceptive disturbance on neural coupling

Elite athletes exhibit superior postural control, a critical component of athletic performance, which is widely attributed to long-term neuroplastic adaptations induced by intensive, task-specific training (Opala-Berdzik et al., 2018). Keller et al. (2018) suggested that improved postural performance in athletes may stem from functional adaptations in both cortical and subcortical structures, particularly during tasks requiring lateral body displacement. Such neural optimization often involves a downregulation of reflexive spinal responses, reducing unnecessary muscle activity and enhancing balance efficiency.

In the present study, elite athletes showed more spatially focused PAC patterns during both bipedal and unilateral stance, whereas controls exhibited broader and less differentiated coupling across frontal, temporal, and parietal areas. This distinction aligns with earlier work suggesting that individuals with extensive balance training may engage neural resources in a more selective manner during postural tasks (Khan et al., 2023). The more focal PAC in athletes, particularly over prefrontal and parietal regions, likely reflects optimized recruitment of attentional and sensorimotor integration networks that have been strengthened through years of balance-specific training. Neuroimaging studies have consistently identified a distributed fronto-parietal network supporting postural regulation (Edwards et al., 2018), and enhanced activity in these regions has been associated with the need to monitor body orientation and resolve sensory uncertainty during stance tasks (Peterson and Ferris, 2018; Stokkermans et al., 2023).

Sensory perturbation during stance is known to influence low- and mid-frequency oscillatory processes (Peterson and Ferris, 2018; Varghese et al., 2014). In the present study, however, the specific cross-frequency coupling patterns differed markedly between tasks and between groups. Rather than exhibiting a single dominant spectral profile, the athlete group showed task-dependent shifts in coupling frequency: delta–gamma interactions (2–32 Hz) emerged during the dual-leg condition, whereas alpha–gamma combinations (10–38 Hz or 10–32 Hz) were most prominent during unilateral stance. These variations indicate that oscillatory coordination in athletes flexibly adapts to the specific postural context rather than following a uniform pattern.

The control group displayed an even broader assortment of phase–amplitude pairings, including delta–gamma (4–22 Hz), alpha–gamma (12–28 Hz; 14–32 Hz), and beta–gamma–range interactions. This diversity, together with the wider spatial distribution of significant PAC sites across frontal, temporal, parietal, and occipital regions, suggests a less selective or more diffuse recruitment of cortical resources during stance regulation. Such variability is consistent with prior evidence that individuals with less balance experience exhibit broader and less differentiated cortical engagement during postural tasks (Hülsdünker et al., 2015). These between-group distinctions suggest differences in how oscillatory activity is organized during stance, but the variability across tasks and surface conditions indicates that PAC likely reflects task-dependent recruitment rather than a uniform control strategy.

During right-leg stance, athletes showed fewer but more spatially focused PAC differences, primarily over frontal regions. This pattern suggests that neural activity during dominant-limb support may be organized in a more concentrated manner, consistent with the localized engagement observed in other stance conditions in this group. The peak PAC at 10 Hz and 32 Hz falls within frequency ranges previously linked to attentional allocation and sensorimotor integration (Fries, 2005). In contrast, controls exhibited broader coupling across frontal, parietal, and occipital areas, echoing the more distributed cortical recruitment reported in individuals with less specialized balance experience (Taubert et al., 2011; Surgent et al., 2019). Within this broader context, the present findings indicate that athletes and controls engage neural oscillatory processes in distinct, task-dependent ways during right-leg stance, consistent with the general pattern observed across other postural conditions.

### 4.2 Single-leg stance and brain lateralization

Single-leg stance introduces asymmetric loading and sensory demands that require coordinated engagement of cortical networks supporting balance regulation. Previous work has shown that unilateral stance elicits lateralized patterns of neural activity, reflecting the distribution of attentional and sensorimotor processing across hemispheres (Chen Y. C. et al., 2021). These task-related asymmetries provide a useful framework for interpreting the hemispheric PAC differences observed in the present study, particularly the distinct contralateral and ipsilateral coupling patterns that varied across stance conditions and groups.

During right-leg stance, both groups showed stronger PAC in the hemisphere contralateral to the supporting limb, consistent with evidence that unilateral stance preferentially engages lateralized cortical processes involved in sensorimotor integration (Muehlbauer et al., 2014). In our data, however, the degree of this contralateral predominance varied with both task conditions and group background. Athletes displayed clearer hemispheric asymmetry on stable surfaces, whereas controls exhibited more pronounced asymmetry when standing on the foam surface. This divergence suggests that lateralized recruitment during dominant-limb stance is not fixed but depends on the interaction between sensory demands and prior balance experience. Rather than reflecting a mechanistic lateralization strategy, the observed patterns may indicate that individuals draw on hemispheric resources differently as task constraints shift (Büchel et al., 2021).

Cortical engagement during single-leg stance has been shown to vary according to limb dominance and task complexity, with unilateral loading eliciting lateralized patterns of neural activity related to sensorimotor processing (Herold et al., 2017; Khan et al., 2023). These findings provide a relevant context for interpreting the hemispheric PAC asymmetries observed in the present study (Lehmann et al., 2020). During right-leg stance, both groups showed stronger contralateral PAC, though the magnitude and surface dependence of this asymmetry differed: athletes demonstrated clearer contralateral predominance on the stable surface, whereas controls showed this pattern mainly under unstable conditions. During left-leg stance, however, athletes exhibited greater ipsilateral PAC in the unstable condition, contrasting with the consistently contralateral pattern observed in controls. These task- and surface-dependent shifts suggest that hemispheric recruitment during unilateral stance varies with both sensory demands and training background, without implying fixed or mechanistic lateralization strategies. The flexibility observed in the athlete group may reflect differences in how oscillatory interactions are organized across hemispheres during postural tasks, consistent with prior evidence that extensive training can influence the distribution of cortical engagement during balance regulation (Del Percio et al., 2008).

The present findings demonstrate that hemispheric PAC patterns during single-leg stance vary systematically with limb and surface conditions. Across both groups, contralateral predominance in PAC was commonly observed, consistent with previous evidence that unilateral stance engages lateralized sensorimotor processes (Velotta et al., 2011; Huurnink et al., 2014). The magnitude of this asymmetry, however, differed by group: athletes showed clearer contralateral coupling during right-leg stance on the stable surface, whereas controls exhibited stronger contralateral dominance on the unstable surface. During left-leg stance, athletes displayed a shift toward ipsilateral PAC under unstable conditions, contrasting with the consistently contralateral pattern in controls. These variations suggest that hemispheric recruitment during unilateral stance is shaped by task demands and prior balance experience, rather than reflecting a single, fixed control pattern.

Traditional center-of-pressure measures capture behavioral aspects of postural asymmetry but provide limited information about the underlying neural processes. In contrast, PAC provides complementary insight into how oscillatory interactions are organized across hemispheres during stance tasks, particularly under conditions with increased proprioceptive demands. The task-dependent lateralization patterns observed here indicate that cortical involvement during SLS may vary across individuals and training backgrounds, supporting the value of incorporating neurophysiological indices when examining postural regulation.

### 4.3 The association between balance performance and phase–amplitude coupling

Phase-amplitude coupling reflects coordinated interactions between low- and high-frequency oscillatory processes and has been linked to sensorimotor integration across a range of behavioral contexts (Canolty and Knight, 2010). In the present study, PAC strength varied across stance tasks and groups, showing task-dependent associations with balance performance rather than a uniform relationship. Notably, clearer negative trends between PAC strength and SE emerged in the stable left- and right-leg conditions, whereas these associations were weaker or inconsistent under unstable conditions. This pattern suggests that CFC-behavior relationships may be more readily expressed when sensory noise is low and postural demands are relatively predictable, allowing oscillatory interactions to align more consistently with behavioral measures of postural stability.

The present findings reveal group differences in the organization of oscillatory activity during postural regulation. Athletes showed PAC patterns that were more distinct across stance conditions, whereas controls exhibited broader but less differentiated coupling. These contrasts suggest that long-term balance experience may influence how oscillatory interactions are recruited during stance. Across tasks, the association between PAC strength and SE varied by condition and was most apparent in stable left- and right-leg stance. In these lower-noise conditions, stronger PAC was accompanied by lower SE in athletes, whereas this pattern was less consistent in controls. The weak or inconsistent PAC-SE associations during foam-surface standing may reflect the increased sensory uncertainty and greater behavioral variability inherent in proprioceptively demanding tasks. Under unstable conditions, subjects rely more heavily on rapid multisensory reweighting and intermittent corrective actions, generating more irregular patterns of motor output (Szczepanski et al., 2014). These rapid adjustments may obscure any systematic relationship between oscillatory coupling and postural variability. Similar dissociations between neural markers and balance metrics under high-noise conditions have been observed in previous studies of postural adaptation and sensory reweighting (Maurer et al., 2006), suggesting that oscillatory signatures may not map onto behavior in a simple linear fashion when the system operates near its stability limits.

Together, these findings reinforce that the functional relevance of PAC during stance cannot be generalized across all postural contexts. Instead, the expression of CFC–behavior associations appears to depend jointly on task constraints and individual balance experience, emerging most clearly when sensory demands are moderate and behavioral variability is low. This task- and experience-dependent organization of oscillatory interactions provides a more nuanced understanding of how neural coupling participates in postural regulation.

### 4.4 Limitations

While our findings offer novel insights into neural coupling mechanisms underlying postural control, several limitations must be acknowledged. First, although the control group exhibited significant behavioral changes across conditions, their coupling strength remained relatively stable. This discrepancy suggests that PAC alone may not fully account for behavioral performance, and other neurophysiological factors-such as sensory feedback processing, motor execution efficiency, or cognitive workload-may also contribute. In addition, this study primarily focused on cortical EEG activity and behavioral measures of balance to characterize the neural mechanisms of postural control. Peripheral physiological data—such as lower-limb electromyography (EMG) or eye-tracking signals reflecting visual attention—were not included, as the experimental design aimed to isolate cortical-level dynamics. Future multimodal studies combining EEG with EMG and eye-tracking could further elucidate the cortico–muscular and visuo–sensorimotor interactions that contribute to adaptive balance regulation.

Second, the observed similarities in coupling strength among non-athletes may reflect alternative control strategies rather than a true absence of neural modulation. It remains unclear whether these patterns represent a baseline neural state or are influenced by task-specific cognitive compensation, individual variability, or insufficient sensitivity of the PAC metric itself. Further work is needed to examine the relative contributions of phase coherence, network modularity, and interregional connectivity.

## 5 Conclusion

The present findings illustrate that phase-amplitude coupling during stance reflects a pattern shaped jointly by task constraints and individual balance experience. PAC did not relate to behavioral performance in a uniform way, and the associations that did emerge appeared only under stable-surface conditions, where postural variability was lower and oscillatory interactions were more consistently expressed. Differences between athletes and non-athletes in both the spatial distribution and spectral structure of PAC further suggest that oscillatory coordination is organized in flexible, context-dependent ways rather than through fixed neural strategies. Future work using longitudinal or multimodal approaches will be essential to clarify the functional significance and potential applied value of these coupling patterns.

## Data Availability

The datasets presented in this article are not readily available because the data supporting the findings of this study are available from the Ethics Committee of Shenyang Sport University and can be made available upon reasonable request. Requests to access the datasets should be directed to chenmingzhu@syty.edu.cn.

## References

[B1] Ahmadi S. Sepehri N. Wu C. Szturm T. (2018). Sample entropy of human gait center of pressure displacement: a systematic methodological analysis. Entropy 20 (8), 579. 10.3390/e20080579 33265668 PMC7513106

[B2] Berger H. (1929). Über das elektroenkephalogramm des menschen. Arch. für Psychiatr. Nervenkrankh. 87 (1), 527–570. 10.1007/bf01797193

[B3] Büchel D. Lehmann T. Ullrich S. Cockcroft J. Louw Q. Baumeister J. (2021). Stance leg and surface stability modulate cortical activity during human single leg stance. Exp. Brain Res. 239, 1193–1202. 10.1007/s00221-021-06035-6 33570677 PMC8068619

[B4] Buzsáki G. Draguhn A. (2004). Neuronal oscillations in cortical networks. Science 304 (5679), 1926–1929. 10.1126/science.1099745 15218136

[B5] Canolty R. T. Knight R. T. (2010). The functional role of cross-frequency coupling. Trends Cognitive Sciences 14 (11), 506–515. 10.1016/j.tics.2010.09.001 20932795 PMC3359652

[B6] Castillo-Barnes D. Gallego-Molina N. J. Formoso M. A. Ortiz A. Figueiredo P. Luque J. L. (2024). Probabilistic and explainable modeling of phase–phase cross-frequency coupling patterns in EEG. Application to dyslexia diagnosis. Biocybern. Biomed. Eng. 44 (4), 814–823. 10.1016/j.bbe.2024.09.003

[B7] Chen X. P. Wang L. J. Chang X. Q. Wang K. Wang H. F. Ni M. (2021). Tai chi and yoga for improving balance on one leg: a neuroimaging and biomechanics study. Front. Neurology 12, 746599. 10.3389/fneur.2021.746599 34721273 PMC8548460

[B8] Chen Y. C. Huang C. C. Zhao C. G. Hwang I. S. (2021). Visual effect on brain connectome that scales feedforward and feedback processes of aged postural system during unstable stance. Front. Aging Neurosci. 13, 679412. 10.3389/fnagi.2021.679412 34366825 PMC8339373

[B9] Cohen M. X. Axmacher N. Lenartz D. Elger C. E. Sturm V. Schlaepfer T. E. (2009). Good vibrations: cross-frequency coupling in the human nucleus accumbens during reward processing. J. Cognitive Neuroscience 21 (5), 875–889. 10.1162/jocn.2009.21062 18702577

[B10] Del Percio C. Rossini P. M. Marzano N. Iacoboni M. Infarinato F. Aschieri P. (2008). Is there a “neural efficiency” in athletes? A high-resolution EEG study. Neuroimage 42 (4), 1544–1553. 10.1016/j.neuroimage.2008.05.061 18602484

[B11] Del Percio C. Babiloni C. Marzano N. Iacoboni M. Infarinato F. Vecchio F. (2009). “Neural efficiency” of athletes’ brain for upright standing: a high-resolution EEG study. Brain Research Bulletin 79 (3-4), 193–200. 10.1016/j.brainresbull.2009.02.001 19429191

[B12] Edwards A. E. Guven O. Furman M. D. Arshad Q. Bronstein A. M. (2018). Electroencephalographic correlates of continuous postural tasks of increasing difficulty. Neuroscience 395, 35–48. 10.1016/j.neuroscience.2018.10.040 30391529

[B13] Engel A. K. Fries P. Singer W. (2001). Dynamic predictions: oscillations and synchrony in top–down processing. Nat. Rev. Neurosci. 2 (10), 704–716. 10.1038/35094565 11584308

[B14] Fries P. (2005). A mechanism for cognitive dynamics: neuronal communication through neuronal coherence. Trends Cognitive Sciences 9 (10), 474–480. 10.1016/j.tics.2005.08.011 16150631

[B15] Henry M. Baudry S. (2019). Age-related changes in leg proprioception: implications for postural control. J. Neurophysiology 122 (2), 525–538. 10.1152/jn.00067.2019 31166819 PMC6734411

[B16] Herold F. Orlowski K. Börmel S. Müller N. G. (2017). Cortical activation during balancing on a balance board. Hum. Movement Science 51, 51–58. 10.1016/j.humov.2016.11.002 27846398

[B49] Hof A. L. (2007). The equations of motion for a standing human reveal three mechanisms for balance[J]. J. biomech. 40 (2), 451–457. 16530203 10.1016/j.jbiomech.2005.12.016

[B17] Horak F. B. Hlavacka F. (2001). Somatosensory loss increases vestibulospinal sensitivity. J. Neurophysiology 86 (2), 575–585. 10.1152/jn.2001.86.2.575 11495933

[B18] Hülsdünker T. Mierau A. Neeb C. Kleinöder H. Strüder H. K. (2015). Cortical processes associated with continuous balance control as revealed by EEG spectral power. Neurosci. Letters 592, 1–5. 10.1016/j.neulet.2015.02.049 25724275

[B19] Huurnink A. Fransz D. P. Kingma I. Hupperets M. D. W. van Dieën J. H. (2014). The effect of leg preference on postural stability in healthy athletes. J. Biomechanics 47 (1), 308–312. 10.1016/j.jbiomech.2013.10.002 24239407

[B20] Hyafil A. Giraud A. L. Fontolan L. Gutkin B. (2015). Neural cross-frequency coupling: connecting architectures, mechanisms, and functions. Trends Neurosciences 38 (11), 725–740. 10.1016/j.tins.2015.09.001 26549886

[B21] Keller J. L. Housh T. J. Smith C. M. Hill E. C. Schmidt R. J. Johnson G. O. (2018). Sex-related differences in the accuracy of estimating target force using percentages of maximal voluntary isometric contractions vs. ratings of perceived exertion during isometric muscle actions. J. Strength & Cond. Res. 32 (11), 3294–3300. 10.1519/JSC.0000000000002210 29176386

[B22] Keyvanara M. Sadigh M. J. Meijer K. Esfahanian M. (2021). A model of human postural control inspired by separated human sensory systems. Biocybern. Biomed. Eng. 41 (1), 255–264. 10.1016/j.bbe.2020.12.008

[B23] Khan H. Qureshi N. K. Yazidi A. Engell H. Mirtaheri P. (2023). Single-leg stance on a challenging surface can enhance cortical activation in the right hemisphere–A case study. Heliyon 9 (2), e13628. 10.1016/j.heliyon.2023.e13628 36846707 PMC9950900

[B24] Kirchner M. Schubert P. Schmidtbleicher D. Haas C. (2012). Evaluation of the temporal structure of postural sway fluctuations based on a comprehensive set of analysis tools. Stat. Mech. Its Appl. 391 (20), 4692–4703. 10.1016/j.physa.2012.05.034

[B25] Koessler L. Maillard L. Benhadid A. Vignal J. P. Felblinger J. Vespignani H. (2009). Automated cortical projection of EEG sensors: anatomical correlation via the international 10–10 system. Neuroimage 46 (1), 64–72. 10.1016/j.neuroimage.2009.02.006 19233295

[B26] Lehmann T. Büchel D. Cockcroft J. Louw Q. Baumeister J. (2020). Modulations of inter-hemispherical phase coupling in human single leg stance. Neuroscience 430, 63–72. 10.1016/j.neuroscience.2020.01.029 32027994

[B27] Malladi R. Johnson D. H. Kalamangalam G. P. Tandon N. Aazhang B. (2018). Mutual information in frequency and its application to measure cross-frequency coupling in epilepsy. IEEE Trans. Signal Processing 66 (11), 3008–3023. 10.1109/tsp.2018.2821627

[B28] Maurer C. Mergner T. Peterka R. J. (2006). Multisensory control of human upright stance. Exp. Brain Research 171 (2), 231–250. 10.1007/s00221-005-0256-y 16307252

[B29] Mierau A. Hülsdünker T. Strüder H. K. (2015). Changes in cortical activity associated with adaptive behavior during repeated balance perturbation of unpredictable timing. Front. Behav. Neurosci. 9, 272. 10.3389/fnbeh.2015.00272 26528154 PMC4604244

[B30] Montesinos L. Castaldo R. Pecchia L. (2018). On the use of approximate entropy and sample entropy with centre of pressure time-series. J. Neuroengineering Rehabilitation 15 (1), 1–15. 10.1186/s12984-018-0465-9 30541587 PMC6291990

[B31] Muehlbauer T. Mettler C. Roth R. Granacher U. (2014). One-leg standing performance and muscle activity: are there limb differences? J. Appl. Biomechanics 30 (3), 407–414. 10.1123/jab.2013-0230 24610423

[B32] Nakata H. Yoshie M. Miura A. Kudo K. (2010). Characteristics of the athletes' brain: evidence from neurophysiology and neuroimaging. Brain Research Reviews 62 (2), 197–211. 10.1016/j.brainresrev.2009.11.006 19944119

[B33] Opala-Berdzik A. Głowacka M. Wilusz K. Kołacz P. Szydło K. Juras G. (2018). Quiet standing postural sway of 10-to 13-year-old,national-level, female acrobatic gymnasts. Acta Bioengineering Biomechanics 20 (2), 117–123. 10.5277/ABB-01060-2017-02 30220710

[B34] Osipova D. Hermes D. Jensen O. (2008). Gamma power is phase-locked to posterior alpha activity. PloS One 3 (12), e3990. 10.1371/journal.pone.0003990 19098986 PMC2602598

[B35] Peterson S. M. Ferris D. P. (2018). Differentiation in theta and beta electrocortical activity between visual and physical perturbations to walking and standing balance. Eneuro 5 (4), ENEURO.0207. 10.1523/ENEURO.0207-18.2018 30105299 PMC6088363

[B36] Richman J. S. Moorman J. R. (2000). Physiological time-series analysis using approximate entropy and sample entropy. Am. J. Physiology-Heart Circulatory Physiology 278 (6), H2039–H2049. 10.1152/ajpheart.2000.278.6.H2039 10843903

[B37] Salimpour Y. Anderson W. S. (2019). Cross-frequency coupling based neuromodulation for treating neurological disorders. Front. Neuroscience 13, 125. 10.3389/fnins.2019.00125 30846925 PMC6393401

[B38] Scheffer-Teixeira R. Tort A. B. L. (2016). On cross-frequency phase-phase coupling between theta and gamma oscillations in the hippocampus. Elife 5, e20515. 10.7554/eLife.20515 27925581 PMC5199196

[B39] Schroeder C. E. Lakatos P. (2009). Low-frequency neuronal oscillations as instruments of sensory selection. Trends Neurosciences 32 (1), 9–18. 10.1016/j.tins.2008.09.012 19012975 PMC2990947

[B40] Slobounov S. Wu T. Hallett M. Shibasaki H. Slobounov E. Newell K. (2006). Neural underpinning of postural responses to visual field motion. Biol. Psychol. 72 (2), 188–197. 10.1016/j.biopsycho.2005.10.005 16338048

[B41] Stokkermans M. Solis-Escalante T. Cohen M. X. Weerdesteyn V. (2023). Midfrontal theta dynamics index the monitoring of postural stability. Cereb. Cortex 33 (7), 3454–3466. 10.1093/cercor/bhac283 36066445 PMC10068289

[B42] Surgent O. J. Dadalko O. I. Pickett K. A. Travers B. G. (2019). Balance and the brain: a review of structural brain correlates of postural balance and balance training in humans. Gait & Posture 71, 245–252. 10.1016/j.gaitpost.2019.05.011 31082657 PMC6594858

[B43] Szczepanski S. M. Crone N. E. Kuperman R. A. Auguste K. I. Parvizi J. Knight R. T. (2014). Dynamic changes in phase-amplitude coupling facilitate spatial attention control in fronto-parietal cortex. PLoS Biology 12 (8), e1001936. 10.1371/journal.pbio.1001936 25157678 PMC4144794

[B44] Taubert M. Lohmann G. Margulies D. S. Villringer A. Ragert P. (2011). Long-term effects of motor training on resting-state networks and underlying brain structure. Neuroimage 57 (4), 1492–1498. 10.1016/j.neuroimage.2011.05.078 21672633

[B45] Tsai Y. Y. Chen Y. C. Zhao C. G. Hwang I. S. (2022). Adaptations of postural sway dynamics and cortical response to unstable stance with stroboscopic vision in older adults. Front. Physiology 13, 919184. 10.3389/fphys.2022.919184 36105297 PMC9465385

[B46] Varghese J. P. Marlin A. Beyer K. B. Staines W. R. Mochizuki G. McIlroy W. E. (2014). Frequency characteristics of cortical activity associated with perturbations to upright stability. Neurosci. Letters 578, 33–38. 10.1016/j.neulet.2014.06.017 24970752

[B47] Velotta J. Weyer J. Ramirez A. (2011). Relationship between leg dominance tests and type of task. ISBS-Conference Proc. Arch.

[B48] Yeh C. H. Lo M. T. Hu K. (2016). Spurious cross-frequency amplitude–amplitude coupling in nonstationary, nonlinear signals. Phys. A Stat. Mech. Its Appl. 454, 143–150. 10.1016/j.physa.2016.02.012 27103757 PMC4834901

